# The repetitive transcranial magnetic stimulation in Alzheimer's disease patients with behavioral and psychological symptoms of dementia: a case report

**DOI:** 10.1186/s12888-023-04864-z

**Published:** 2023-05-23

**Authors:** Zhen Yang, Ying Zhou

**Affiliations:** 1 University of South China, Heng Yang, 421001 China; 2 Neurology Department, The First Hospital Of Chang Sha, Chang Sha City, 410000 China

**Keywords:** Alzheimer’s disease, Repeated transcranial magnetic stimulation, Behavioral and psychological symptoms of dementia, Case report

## Abstract

**Background:**

Repetitive transcranial magnetic stimulation is a noninvasive intervention, can significantly reduce behavioral and psychological symptoms and cognitive impairment in AD patients. Only few cases have been reported the adverse reactions after the treatment. This report described the different adverse reactions after repetitive transcranial magnetic stimulation with different parameters.

**Patient presentation:**

This article reports a patient with dementia complicated with mental behavior disorder who was treated with repetitive transcranial magnetic stimulation (rTMS) in spite of poor drug response. First, 1 Hz rTMS was initiated. After 1 month, the patient showed improved symptoms of mental behavior, decreased cognitive function and prolonged sleep duration. After switched to 10 Hz rTMS, the patient’s cognitive function and mental behavior abnormalities improved, and the sleep time returned to normal. However, epilepsy occurred after one session and was changed to 0.8 Hz rTMS treatment. The patient’s symptoms improved and did not have seizure.

**Conclusion:**

Repetitive transcranial magnetic stimulation has a positive effect on cognitive function and Behavioral And Psychological Symptoms Of Dementia, and adverse reactions are inevitable. Playing personalized treatment according to the patients can reduce occurrence of adverse reactions.

## Introduction

Alzheimer’s disease (AD) is a progressive neurodegenerative disease that seriously endangers the health of middle-aged and elderly people. With the ageing of the population, the number of people suffering from AD has increased rapidly, with a new patient every three seconds, which is expected to increase by three times by 2050, bring a heavy burden to families and society [1]. In addition to cognitive impairment, dementia patients also show non-recognized neuropsychiatric symptoms, which seriously affect the quality of life of patients and caregivers. It is estimated that almost all people with dementia show at least one or more symptoms of psychological or behavioral disorders during the disease development. About 50% of AD and other dementia patients develop into psychosis that is primarily manifested by hallucinations and delusions [2].

Behavioral and psychological symptoms of dementia (BPSD) refer to a series of symptoms often occurring in dementia patients, including apathy, depression, mood disorders, anxiety, hallucinations, mental illness, restlessness, sleep disorders, etc. [3, 4]. At present, for patients with dementia, especially patients with BPSD, treatment methods are limited, and the treatment effect is not satisfactory [5, 6]. rTMS (repetitive transcranial magnetic stimulation), as a noninvasive intervention, has developed into a promising option for the treatment and rehabilitation of neuropsychiatric disorders [7]. Due to the variety of BPSD manifestations, patients can be combined with several different manifestations, and the efficacy of drug therapy is limited and some patients are prone to severe adverse reactions. Non-drug treatment has become the important choice for the treatment of BPSD [8], including dietary treatments (polyphenols) [9], complementary and alternative treatments(Shiatsu) [10] and physical therapy(rTMS, transcranial direct current stimulation). Now, some studies apply rTMS to treating cognitive impairment of dementia [11–13] and BPSD [13–15]. Transcranial magnetic stimulation can enhance or decrease the cortical excitability by transmitting magnetic pulses to cortex [16–18]. The excitatory or inhibitory effects of rTMS are hypothesized to be long-term potentiation (LTP)-like and long-term depression (LTD)-like. LTP and LTD are two mechanisms of synaptic plasticity that involve several biological phenomena and ultimately lead to synaptic strengthening (LTP) or weakening (LTD), that is the increase or decrease of synaptic efficiency [19]. rTMS can induce and regulate metaplasticity. Metaplasticity refers to the activity-dependent modulation of synaptic plasticity. However, understanding of the cellular and molecular mechanisms underlying different forms of synaptic plasticity, including metaplasticity, remains limited [20]. In this article, we report a case of rTMS in the treatment of dementia with BPSD. The patient’s some symptoms improved, and new problems at the same time emerged. We adjusted the treatment to solve new problem. A systematic literature search provided no previous published reports about using rTMS to treat the AD patient with BPSD.

## Case report

The patient is a 73-year-old woman with a senior high school education. In December 2019, she usually lost everything, cooked in the wrong order, forgot where things were put. Patient and their families do not pay attention to this phenomenon. The patient’s cognitive function continued to decline, gradually she started to dress incorrectly and can’t recognize familiar people and she can’t go home on her own. In February 2020, the patient showed behavioral and psychological symptoms of dementia, such as abnormal restlessness at night, abnormal noise during the day, beating and abusing family members, irritability, gibberish, unexplained fear and crying, delusion, auditory hallucinations, agitation and other phenomena.

**Fig. 1:**
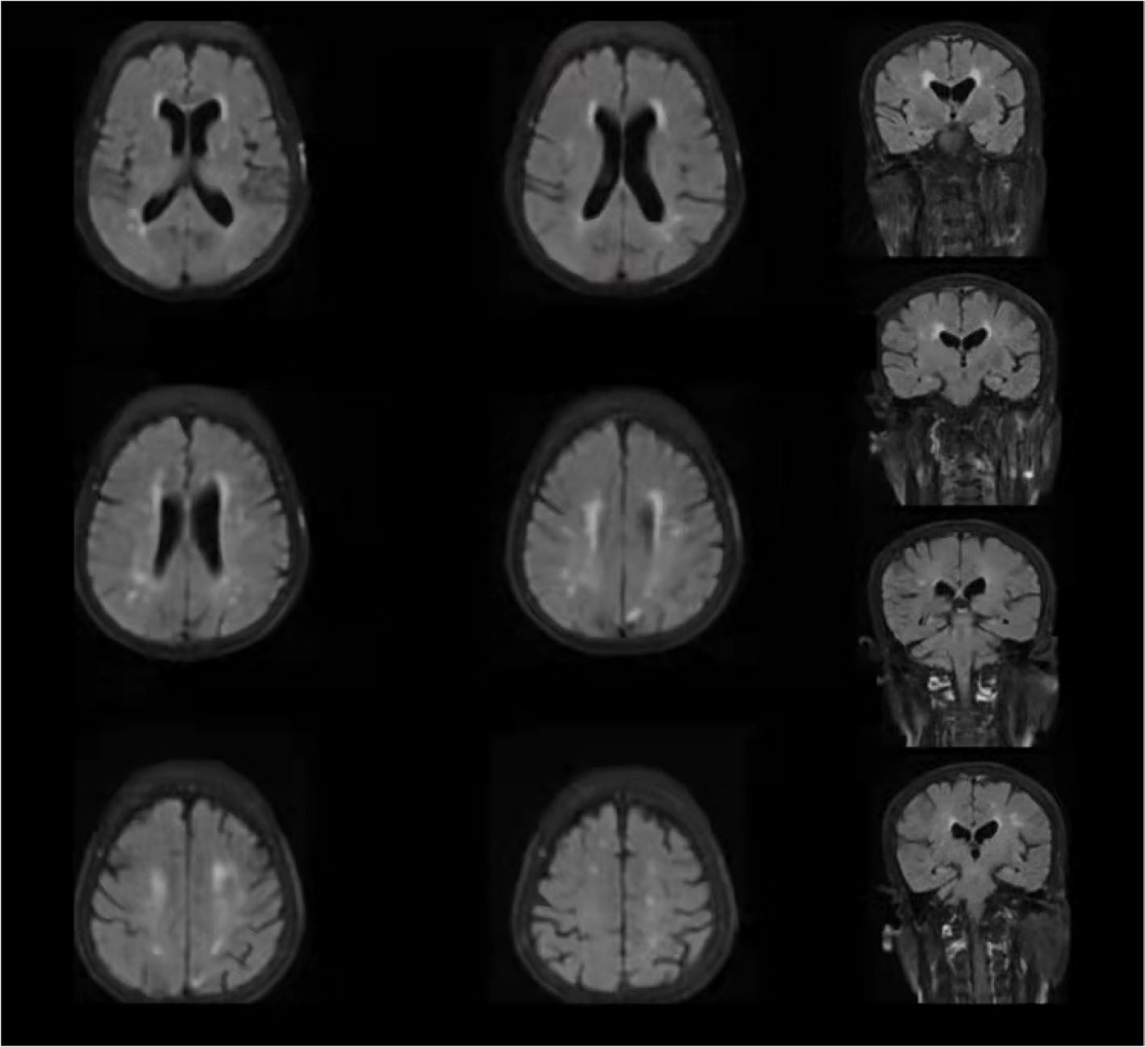
The patient’s MRI results

**Fig. 2:**
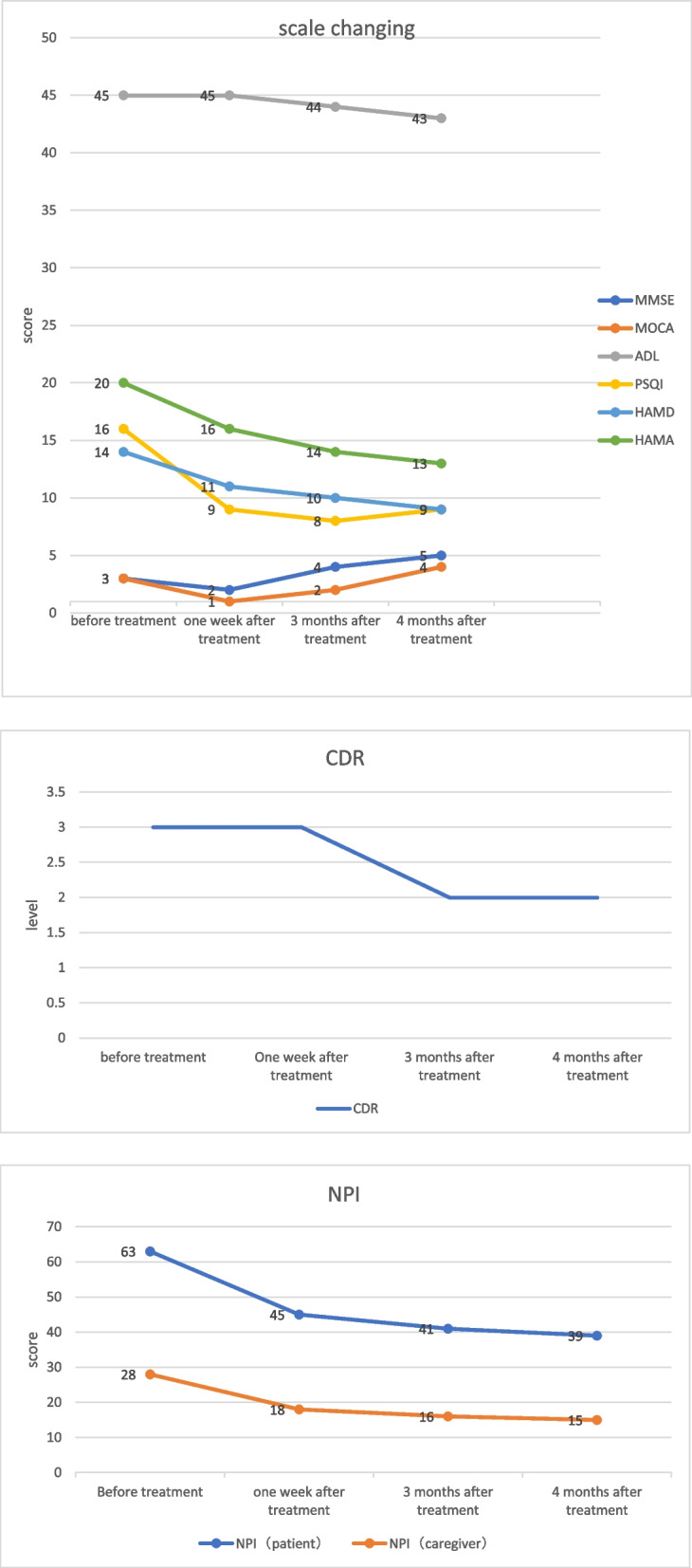
Chang in scale. MMSE, Mini-mental state examination. MOCA, Montreal cognitive assessment. NPI, Neuropsychiatric inventory. ADL Activity of daily living scale. HAMD, Hamilton Depression Scale. HAMA, Hamilton anxiety scale. PSQI, Pittsburgh sleep quality index. CDR, Clinical Dementia Rating Scale

The patient was started on Donepezil hydrochloride (5 mg q.d.). Due to persistent chest tightness, the patient returned to the hospital the day after, and the treatment was switched to memantine hydrochloride (5 mg q.d.) and antipsychotics (risperidone 4 mg q.d.). We inform families about some considerations for caring for patients, including environmental management (trying to guide or place the patient in a relatively spacious environment, avoiding bright light or loud sounds), and reassurance (appropriate speech and body expression).The patient's symptoms did not improve. Therefore, the patient was hospitalized in our hospital for treatment and improved the examination. Investigations were completed with blood tests, which were in normal range. MRI scan of the brain (Fig. 1) showed subcortical ischemia, leukoaraiosis and bilateral hippocampal atrophia. The scale results are shown in Fig. 2. Clinical Dementia Rating Scale (CDR) showed Level 3. Hachinski ischemic score was 4. AD was considered for clinical diagnosis. Meanwhile, we should pay attention to the condition of subcortical ischemia in patients. Vascular damage may affect the selection of rTMS parameters [21].

Patient was treated with rTMS on the basis of maintenance medication (Memantine Hydrochloride + Risperidone). The rTMS protocol was carried out using a medical device targeting the bilateral dorsolateral prefrontal cortex (DLPFC). The stimulations parameters were: 1 Hz frequency, 86% of resting motor threshold (rMT), 30 trains, 60 pulses per train, 3 s intertrain interval, and 1075 pulses per session. The subject received 1 daily sessions for the first consecutive 5 days of treatment (5 sessions), and then 2 weekly sessions for the next 3 weeks. Risperidone was stopped after 3 treatments, because of the improvement in the behavioral and psychological symptoms. After the total treatment, the patient’s mental and behavioral abnormalities improved, and her family expressed that the patient did not appear delusions, auditory hallucinations and other manifestations. And the patient was no longer noisy at night, could sleep quietly, and could recognize the family members, could help her husband to do simple house work.

However, cognitive improvement did not continue, and even returned to the original level, and there was an extension of sleep duration, lasting up to 10–20 h a day during 3 months after treatment. We then switched to 10 Hz rTMS of the left DLPFC (80%rMT, 15 trains, 15 pulses per train, 12 s intertrain interval, and 795 pulses per session) and 1 Hz rTMS of the right DLPFC (80%rMT, 25 trains, 25 pulses per train, 3 s intertrain interval, and 800 pulses per session), with 8 times in total, once a week. After this treatment, the cognitive and mental behavior abnormalities of the patients improved compared with previous ones, and the sleep time returned to normal (6–8 h every night).

**Fig. 3:**
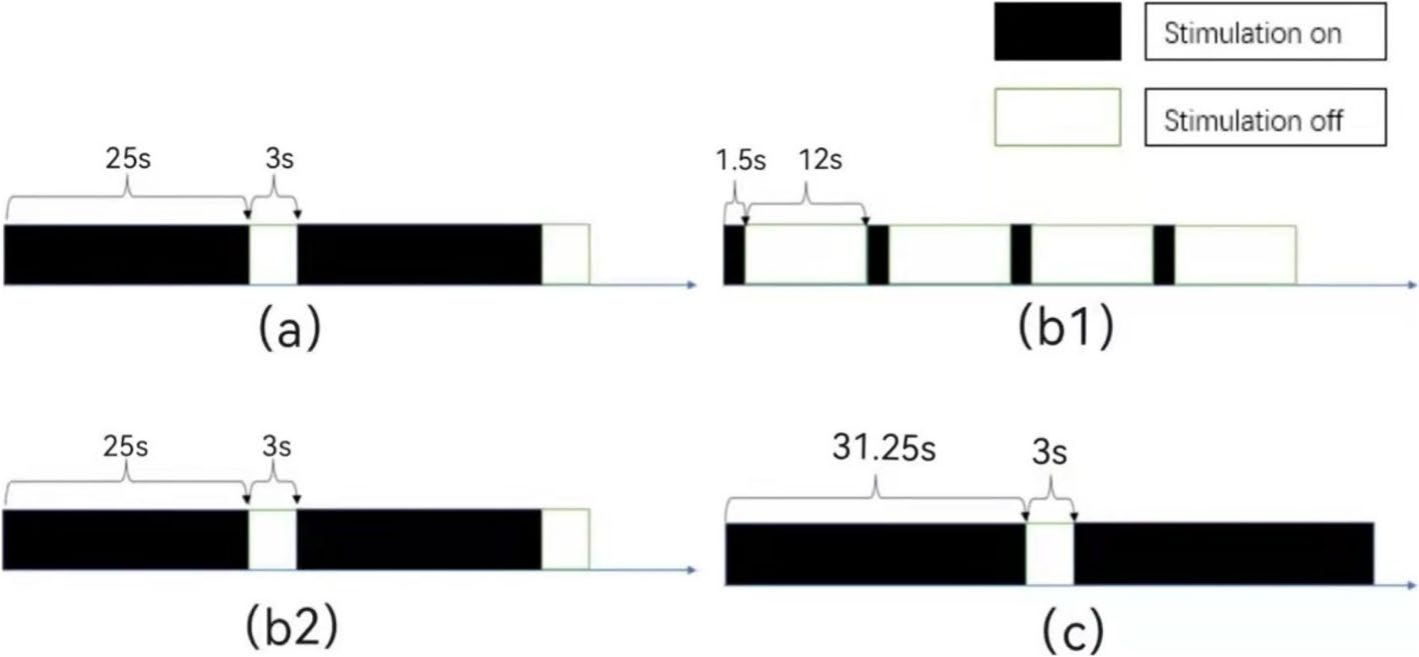
**a** the first stimulation pattern (1 Hz bilateral DLPFC). **b1** the second stimulation pattern (10 Hz left DLPFC). **b2** the second stimulation pattern (1 Hz right DLPFC). **c** the third stimulation pattern (0.8 Hz right DLPFC)

After a rTMS treatment, the patient developed an epileptic seizure, presenting as a generalized tonic–clonic seizures and a disturbance of consciousness. The symptoms persisted for several minutes and self-relieved. The 0.8 Hz rTMS of the right DLPFC (80%rMT, 25 trains, 25 pulses per train, 3 s intertrain interval, and 800 pulses per session) were used in the patient with total 5times, once a week. Ambulatory Electroencephalogram (EEG) was normal. After several months of follow-up, the patient did not develop epileptic seizures and the BPSD and cognitive function improved. The course of treatment is shown in Table 1 and Fig. 3. The scale changing is shown in Fig. 2.

**Table 1:** The course of treatment

	Main problem	Method	Result
First	Mental behavior disorder and reducing cognitive function	1 Hz bilateral DLPFC 86%MT, 1075 total pulses, 30 stimulation, 25 s stimulation times, 3 s interval	BPSD (delusion, auditory hallucinations, agitation) and cognitive function improved, Prolonged sleep
Second	Prolonged sleep and reducing cognitive function	10 Hz left DLPFC 80%MT, 795 total pulses,15 stimulation, 1.5 s stimulation times, 12 s interval 1 Hz right DLPFC, 80%MT, 800 total pulses, 25 stimulation, 25 s stimulation times, 3 s interval	BPSD and cognitive function improved, Sleep time become normal Epileptic attack
Third	Epileptic seizure	0.8 Hz right DLPFC, 80%MT, 800 total pulses, 25 stimulation, 31.25 s stimulation times, 3 s interval	BPSD and cognitive function improved, Epilepsy has not occurred

## Discussion

This case reports a patient with sever Alzheimer’s disease complicated with BPSD treated with rTMS. We reviewed the previous research. Most research shown positive effect in the AD patient with BPSD, but there was no development of a unified treatment plans. The adverse action after treatment is rarely mentioned. For this reason, we report this case to share some experience.

According to the clinical evaluation, the patient had severe dementia combined with multiple BPSD manifestations. In addition to severe cognitive decline, the patient was associated with sleep disorders, depression, irritability, delusion, auditory hallucination, anxiety and other BPSD manifestations. Other related factors (drugs, infection, environment) were excluded, and the patient’s mental and behavioral abnormalities were thought to be associated with cognitive deterioration. The primary goal was to improve the symptom of BPSD on the basis of medication. Some meta-analysis suggested that rTMS was capable of persistently improving the behavioral and psychological outcomes at least for weeks to months [22, 23]. Therefore, the treatment method for this patient was 1 Hz, bilateral DLPFC. After treatment, the patient’s cognitive function and BPSD were improved compared with the previous state. After a short time, the patient’s cognitive function returned to the state before treatment, and there was a prolonged sleep, but auditory hallucination, delusions, anxiety and irritability improved. In clinical trials, bilateral low-frequency rTMS in the DLPFC has found to relieve the symptoms of generalized anxiety disorder (GAD). The mechanism may be related with increased brain-derived neurotrophic factor (BDNF) levels and 5-hydroxytryptamine (5-HT) release in the brain, and low-frequency rTMS also are equally effective for symptoms such as auditory hallucination and delusions [24, 25]. DLPFC promote the formation of long-term memory by interacting with areas in the medial temporal lobe network (such as hippocampus) through the memory coding process [2]. Low-frequency rTMS can directly hyperpolarize DLPFC nerve cells through pulsed magnetic field, and inhibit the hyperexcitation (high arousal) state of the cerebral cortex. At the same time, BDNF and its high-affinity Tyrosine Kinase receptor B (TrkB) play an important role in supporting the survival of various neuron population in central nervous system disease (CNS), which help to protect neurons from neurodegenerative diseases, including AD [26–28]. Moreover, according to previous AD animal experiments, rTMS reversed the loss of hippocampal nerve growth factor and BDNF that induced by amyloidβ-protein 42(Aβ42), increased the affinity of BDNF to TrkB in the prefrontal cortex, enhanced hippocampal LTP, and reduced hippocampal β-amyloid precursor protein(APP) [29–31]. In fact, studies have shown that the amyloidβ-protein cortical load is affected by the sleep–wake cycle and is not associated with plaque formation [32]. Most studies about insomnia have shown the efficacy of LF-rTMS, and low-frequency rTMS targeting the DLPFC can improve sleep structure and reduce overarousal [33]. In addition, forced sleep reduced Aβ burden and associated APP-dependent synaptic abnormalities [34].

The patient decreased again after a brief improvement in cognition, and the symptoms of BPSD improved compared with the previous one, like auditory hallucination, delusions and irritability, but the sleep was prolonged. The change might be related with prolonged sleep. The patient who has not used drugs for affecting sleep, is currently considered as a result of low frequency rTMS reduction in cortical excitability. And there was a brief improvement in cognitive function during treatment. The reason for the decline may be related to the weakening or disappearance of rTMS induced LTP or LTD-like effect, and the effect of low frequency rTMS on patients with depressive symptoms is controversial. Previous studies have shown that depression may be a precursor to dementia or that these two diseases from common pathophysiology are interactive [27]. The most common approach to treating depression with rTMS is to use standard high-frequency 10 Hz TMS in the left prefrontal cortex [35]. rTMS was applied to the prefrontal cortex and induced magnetic fields to depolarize potential neurons and modulate neural circuits involved in mood regulation and depressive symptoms [35]. The treatment plan was changed to use high frequency (HF) rTMS targeting the left DLPFC and low frequency (LF) rTMS targeting right DLPFC. High frequency TMS improved cognition better than low frequency [36], and high frequency TMS of left DLPFC and low frequency rTMS of right DLPFC significantly improved memory function [37]. The excitatory effect induced by high frequency rTMS counteracts the inhibitory effect of sleep prolongation in patients.

The patient developed epilepsy after a rTMS treatment. The most serious adverse event of rTMS is the induction of epilepsy, which is rare but occurs most frequently in high frequency TMS [38]. Its occurrence is related to the diffusion of neuronal excitement to the motor cortex by directly stimulating the motor cortex or stimulating the adjacent brain regions [39]. Reducing the cortical excitability of seizure focal points can reverse or counteract the hyperexcitability of epileptic foci, leading to a reduction in seizure frequency [40]. So LF-rTMS over left DLPFC was used in the patient. It can be concluded from the above that rTMS treatment has a positive effect on patients' recognition and BPSD symptoms. Due to the different symptoms and pathogenesis of patients, different adverse reactions may occur, and a large number of clinical trials are still needed to draw further conclusions to explore how to design individualized plan to effectively avoid adverse risks and enable patients to gain more benefits from treatment.

## Conclusion

Alzheimer's disease (AD) has become a major problem of global public health and social sustainable development. BPSD is associated with cognitive deterioration and an accelerated period of dementia, with greater personal distress and caregiver burden, increased risk of complications such as falls and fractures, and higher treatment and care costs. rTMS has been used in clinical practice as a treatment with high safety and less side effects, which can reduce the use of anti-psychotic drugs to reduce the side effect of the drugs. Many studies have also shown that this approach is effective in patients' memory, mood, personality and behavior changes. Different Alzheimer's patients have different clinical manifestations, and most of them are complicated with more or less other diseases. How to make personalized treatment planning and reduce the occurrence of adverse reactions requires exploration of the more essential pathogenesis of patients and skilled application of rTMS.

## Data Availability

Data sharing is not applicable to this article as no datasets were generated or analysed.

## Abbreviations

AD: Alzheimer’s disease
BPSD: Behavioral and psychological symptoms of dementia
rTMS: Repetitive transcranial magnetic stimulation
MRI: Magnetic Resonance Imaging
LTP: Long-term potentiation
LTD: Long-term depression
MTA: Medial temporal lobe atrophy
CDR: Clinical Dementia Rating Scale
MMSE: Mini-mental state examination
MOCA: Montreal cognitive assessment
NPI: Neuropsychiatric inventory
ADL: Activity of daily living scale
HAMD: Hamilton Depression Scale
HAMA: Hamilton anxiety scale
PSQI: Pittsburgh sleep quality index
DLPFC: Dorsolateral prefrontal cortex
AMPAR: α-Amino-3-hydroxy-5-methyl-4-isoxazole-propionic acid receptor
rMT: Resting motor threshold
NMDAR: N-methyl-D-aspartic acid receptor
GAD: Generalized anxiety disorder
BDNF: Brain-derived neurotrophic factor
5-HT: 5-Hydroxytryptamine
GABA: γ-Aminobutyric acid
TrkB: Tyrosine Kinase receptor B
CNS: Central nervous system disease
Aβ: Amyloidβ-protein
APP: β-Amyloid precursor protein
LF: Low frequency
HF: High frequency

## References

[1] O'Shaughnessy NJ, Chan JE, Bhome R, Gallagher P, Zhang H, Clare L, Sampson EL, Stone P, Huntley J (2021). Awareness in severe Alzheimer's disease: a systematic review. Aging Ment Health.

[2] Bucki A, Marcinkowska M, Śniecikowska J, Zagórska A, Jamrozik M, Pawłowski M, Głuch-Lutwin M, Siwek A, Jakubczyk M, Pytka K, Jastrzębska-Więsek M, Partyka A, Wesołowska A, Mierzejewski P, Kołaczkowski M (2020). Multifunctional 6-fluoro-3-[3-(pyrrolidin-1-yl)propyl]-1,2-benzoxazoles targeting behavioral and psychological symptoms of dementia (BPSD). Eur J Med Chem.

[3] Lyketsos CG, Steinberg M, Tschanz JT, Norton MC, Steffens DC, Breitner JC (2000). Mental and behavioral disturbances in dementia:findings from the Cache County Study on memory in aging. Am JPsychiatry.

[4] Devshi R, Shaw S, Elliott-King J, Hogervorst E, Hiremath A, Velayudhan L, Kumar S, Baillon S, Bandelow S (2015). Prevalence of behavioural and psychological symptoms of dementia in individuals with learning disabilities. Diagnostics (Basel).

[5] Jin B, Liu H (2019). Comparative efficacy and safety of therapy for the behavioral and psychological symptoms of dementia: a systemic review and Bayesian network meta-analysis. J Neurol.

[6] Livingston G, Johnston K, Katona C, Paton J, Lyketsos CG (2005). Systematic review of psychological approaches to the management of neuropsychiatric symptoms of dementia. Am J Psychiatry.

[7] Sabbagh M, Sadowsky C, Tousi B, Agronin ME, Alva G, Armon C, Pascual-Leone A (2019). Effects of a combined transcranial magnetic stimulation (TMS) and cognitive training intervention in patients with Alzheimer's disease. Alzheimers Dement.

[8] Calsolaro V, Femminella GD, Rogani S, Esposito S, Franchi R, Okoye C, Rengo G, Monzani F (2021). Behavioral and Psychological Symptoms in Dementia (BPSD) and the Use of Antipsychotics. Pharmaceuticals (Basel).

[9] Caruso G, Godos J, Privitera A, Lanza G, Castellano S, Chillemi A, Bruni O, Ferri R, Caraci F, Grosso G (2022). Phenolic Acids and Prevention of Cognitive Decline: Polyphenols with a Neuroprotective Role in Cognitive Disorders and Alzheimer's Disease. Nutrients.

[10] Lanza G, Centonze SS, Destro G, Vella V, Bellomo M, Pennisi M, Bella R, Ciavardelli D (2018). Shiatsu as an adjuvant therapy for depression in patients with Alzheimer's disease: A pilot study. Complement Ther Med.

[11] Ahmed MA, Darwish ES, Khedr EM, El Serogy YM, Ali AM (2012). Effects of low versus high frequencies of repetitive transcranial magnetic stimulation on cognitive function and cortical excitability in Alzheimers Dement. J Neurol.

[12] Lee J, Choi BH, Oh E, Sohn EH, Lee AY (2016). Treatment of Alzheimer’s disease with repetitive transcranial magnetic stimulation combined with cognitive training: a prospective, randomized, double-blind, placebo-controlled study. J Clin Neurol.

[13] Zhang F, Qin Y, Xie L, Zheng C, Huang X, Zhang M (2019). High-frequency repetitive transcranial magnetic stimulation combined with cognitive training improves cognitive function and cortical metabolic ratios in Alzheimer’s disease. J Neural Transm.

[14] Suemoto CK, Apolinario D, Nakamura-Palacios EM, Lopes L, Leite RE, Sales MC, Nitrini R, Brucki SM, Morillo LS, Magaldi RM, Fregni F (2014). Effects of a non-focal plasticity protocol on apathy in moderate Alzheimer’s disease: a randomized, double-blind, sham-controlled trial. Brain Stimul.

[15] Wu Y, Xu W, Liu X, Xu Q, Tang L, Wu S (2015). Adjunctive treatment with high frequency repetitive transcranial magnetic stimulation for the behavioral and psychological symptoms of patients with Alzheimer's disease: a randomized, double-blind, sham-controlled study. Shanghai Arch Psychiatry.

[16] Ferrucci R, Mrakic-Sposta S, Gardini S, Ruggiero F, Vergari M, Mameli F, Arighi A, Spallazzi M, Barocco F, Michelini G, Pietroboni AM, Ghezzi L, Fumagalli GG, D’Urso G, Caffarra P, Scarpini E, Priori A, Marceglia S (2018). Behavioral and neurophys-iological effects of transcranial direct current stimulation (tDCS) infronto-temporal dementia. Front Behav Neurosci.

[17] Hsu WY, Ku Y, Zanto TP, Gazzaley A (2015). Effects of noninva-sive brain stimulation on cognitive function in healthy aging and Alzheimer’s disease: a systematic review and meta-analysis. Neurobiol Aging.

[18] Ni Z, Chen R (2015). Transcranial magnetic stimulation to under-stand pathophysiology and as potential treatment for neurodegen-erative diseases. Transl Neurodegener.

[19] Valero-Cabré A, Amengual JL, Stengel C, Pascual-Leone A, Coubard OA (2017). Transcranial magnetic stimulation in basic and clinical neuroscience: A comprehensive review of fundamental principles and novel insights. Neurosci Biobehav Rev.

[20] Cantone M, Lanza G, Ranieri F, Opie GM, Terranova C (2021). Editorial: Non-invasive Brain Stimulation in the Study and Modulation of Metaplasticity in Neurological Disorders. Front Neurol..

[21] Cantone M, Lanza G, Fisicaro F, Pennisi M, Bella R, Di Lazzaro V, Di Pino G (2020). Evaluation and Treatment of Vascular Cognitive Impairment by Transcranial Magnetic Stimulation. Neural Plast.

[22] Wang X, Mao Z, Yu X (2020). The role of noninvasive brain stimulation for behavioral and psychological symptoms of dementia: a systematic review and meta-analysis. Neurol Sci.

[23] Gu L, Xu H, Qian F (2022). Effects of Non-Invasive Brain Stimulation on Alzheimer's Disease. J Prev Alzheimers Dis.

[24] Lu R, Zhang C, Liu Y, Wang L, Chen X, Zhou X (2018). The effect of bilateral low-frequency rTMS over dorsolateral prefrontal cortex on serum brain-derived neurotropic factor and serotonin in patients with generalized anxiety disorder. Neurosci Lett.

[25] Sommer IE, Slotema CW, Daskalakis ZJ, Derks EM, Blom JD, van der Gaag M (2012). The treatment of hallucinations in schizophrenia spectrum disorders. Schizophr Bull.

[26] Guo W, Nagappan G, Lu B (2018). Differential effects of transient and sustained activation ofBDNF-TrkB signaling. Dev Neurobiol.

[27] Sampaio TB, Savall AS, Gutierrez MEZ, Pinton S (2017). Neurotrophic factors in Alzheimer’s and Parkinson’s diseases: Implications for pathogenesis and therapy. Neural Regen Res.

[28] Mitre M, Mariga A, Chao MV (2017). Neurotrophin signalling: Novel insights into mechanisms and pathophysiology. Clin Sci.

[29] SoundaraRajan T (2017). Mechanism of action for rTMS: A working hypothesis based on animal studies. Front Physiol.

[30] Wang F (2015). Improvement of spatial learning by facilitating large-conductance calcium-activated potassium channel with tran-scranial magnetic stimulation in Alzheimer’s disease model mice. Neuropharmacology.

[31] Huang Z, Tan T, Du Y, Chen L, Fu M, Yu Y, Zhang L, Song W, Dong Z (2017). Low-Frequency Repetitive Transcranial Magnetic Stimulation Ameliorates Cognitive Function and Synaptic Plasticity in APP23/PS45 Mouse Model of Alzheimer's Disease. Front Aging Neurosci.

[32] Lanza G, DelRosso LM, Ferri R (2022). Sleep and homeostatic control of plasticity. Handb Clin Neurol.

[33] Feng J, Zhang Q, Zhang C, Wen Z, Zhou X (2019). The Effect of sequential bilateral low-frequency rTMS over dorsolateral prefrontal cortex on serum level of BDNF and GABA in patients with primary insomnia. Brain Behav.

[34] Lanza G, Fisicaro F, Cantone M, Pennisi M, Cosentino FII, Lanuzza B, Tripodi M, Bella R, Paulus W, Ferri R (2023). Repetitive transcranial magnetic stimulation in primary sleep disorders. Sleep Med Rev.

[35] McClintock SM, Reti IM, Carpenter LL, McDonald WM, Dubin M, Taylor SF, Cook IA, O'Reardon J, Husain MM, Wall C, Krystal AD, Sampson SM, Morales O, Nelson BG, Latoussakis V, George MS, Lisanby SH, National Network of Depression Centers rTMS Task Group, American Psychiatric Association Council on Research Task Force on Novel Biomarkers and Treatments (2018). Consensus Recommendations for the Clinical Application of Repetitive Transcranial Magnetic Stimulation (rTMS) in the Treatment of Depression. J Clin Psychiatry.

[36] Wang X, Mao Z, Ling Z, Yu X (2020). Repetitive transcranial magnetic stimulation for cognitive impairment in Alzheimer's disease: a meta-analysis of randomized controlled trials. J Neurol.

[37] Chou YH, Ton That V, Sundman M (2020). A systematic review and meta-analysis of rTMS effects on cognitive enhancement in mild cognitive impairment and Alzheimer's disease. Neurobiol Aging..

[38] Tsuboyama M, Kaye HL, Rotenberg A (2020). Review of Transcranial Magnetic Stimulation in Epilepsy. Clin Ther.

[39] Rossi S, Hallett M, Rossini PM (2009). Safety of TMS Consensus Group. Safety, ethical considerations, and application guidelines for the use of transcranial magnetic stimulation in clinical practice and research. Clin Neurophysiol.

[40] VanHaerents S, Chang BS, Rotenberg A, Pascual-Leone A, Shafi MM (2020). Noninvasive Brain Stimulation in Epilepsy. J Clin Neurophysiol.

